# *CSE1L*, *DIDO1* and *RBM39* in colorectal adenoma to carcinoma progression

**DOI:** 10.1007/s13402-012-0088-2

**Published:** 2012-06-19

**Authors:** Anke H. Sillars-Hardebol, Beatriz Carvalho, Jeroen A.M. Beliën, Meike de Wit, Pien M. Delis-van Diemen, Marianne Tijssen, Mark A. van de Wiel, Fredrik Pontén, Gerrit A. Meijer, Remond J. A. Fijneman

**Affiliations:** 1 Department of Pathology, VU University Medical Center, CCA1.08, PO Box 7057, 1007 MB Amsterdam, The Netherlands; 2 Department of Pathology, VU University Medical Center, PO Box 7057, 1007 MB Amsterdam, The Netherlands; 3 Department of Epidemiology and Biostatistics, VU University Medical Center, Amsterdam, The Netherlands; 4 Department of Mathematics, VU University, Amsterdam, The Netherlands; 5 Department of Genetics and Pathology, The Rudbeck Laboratory, Uppsala University, Uppsala, Sweden

**Keywords:** Colorectal adenoma-to-carcinoma progression, Chromosome 20q gain, *CSE1L*, *DIDO1*, *RBM39*, Driver genes

## Abstract

**Background:**

Gain of chromosome 20q is an important factor in the progression from colorectal adenomas to carcinomas. Genes that drive 20q gain are expected to show correlation of mRNA and protein expression levels with 20q DNA copy number status while functionally influencing cancer processes. *CSE1L*, *DIDO1* and *RBM39* are located on the 20q amplicon and affect processes such as cell viability and anchorage-independent growth in colorectal cancer. This study aimed to investigate whether *CSE1L*, *DIDO1* and *RBM39* may drive 20q amplification.

**Methods:**

Protein expression levels were examined by immunohistochemical evaluation of tissue microarrays containing a series of colorectal adenoma and carcinoma samples, which were characterized by genome-wide (microarray-based) DNA and mRNA profiling.

**Results:**

*CSE1L*, *DIDO1* and *RBM39* mRNA expression levels correlated with chromosome 20q DNA copy number status. CSE1L protein expression was not associated with 20q gain, although its expression was increased in carcinomas compared to adenomas. DIDO1 and RBM39 protein expression was quite strong in the majority of tumors irrespective of 20q DNA copy number status.

**Conclusion:**

The lack of correlation between protein expression levels and 20q DNA copy number status implies that *CSE1L*, *DIDO1* and *RBM39* are merely passengers rather than drivers of chromosome 20q gain in colorectal adenoma-to-carcinoma progression.

**Electronic supplementary material:**

The online version of this article (doi:10.1007/s13402-012-0088-2) contains supplementary material, which is available to authorized users.

## Introduction

Colorectal cancer (CRC) is the second cause of cancer related deaths in the western world [1]. Worldwide CRC accounts for 600,000 deaths annually. CRC development from normal colon epithelium is initiated by disruption of the WNT signalling pathway which gives rise to benign precursor lesions, i.e. adenomas. Colorectal adenomas can progress into CRC, which is estimated to take place in about 5 % of cases [2]. CRC carcinogenesis is driven by multiple genetic alterations, which accumulate in a developing tumor upon introduction of genomic instability. Microsatellite instability (MSI) is caused by failure of the DNA mismatch repair system and leads to accumulation of DNA mutations in approximately 15 % of CRCs. Chromosomal instability (CIN) is observed in the majority of CRCs (about 85 %) and is characterized by gains and losses of chromosomal regions. These gross aberrations lead to deletion or amplification of tumor suppressor genes, oncogenes, and/or noncoding RNAs such as miRNAs, resulting in aberrant expression of genes that affect cancer-related biological processes [3–5].

Gain of chromosomal region 20q is frequent in CRC; it is present in more than 60 % of carcinomas [6, 7]. Several functional consequences of chromosome 20q gain have been reported; 20q gain has been associated with colorectal adenoma-to-carcinoma progression [7] and it correlates with poor prognosis in CRC patients [8]. Usually, large fragments of the 20q arm are amplified as a whole, suggesting a role of multiple 20q genes on CRC progression. Recently, we confirmed by functional analyses that multiple genes on chromosome 20q contribute to cancer processes [9]. *AURKA* and *TPX2* were identified as genes that promote 20q gain-driven colorectal adenoma-to-carcinoma progression. However, other genes located on 20q also contributed to cancer-related processes, including *CSE1L*, *DIDO1* and *RBM39*. *CSE1L* and *RBM39* affect cell viability while *CSE1L* and *DIDO1* contribute to anchorage-independent growth (Suppl. Fig. 1) [9]. Therefore, these genes may be drivers of 20q gain associated tumor progression.

Chromosomal regions that frequently show increased copy numbers in tumors are believed to drive carcinogenesis by affecting mRNA and protein expression levels of relevant ‘driver’ genes. Therefore, genes whose mRNA and protein expression is influenced by its DNA copy number status are candidate genes that may drive the gain of the amplicon. From previous studies it is known that increased mRNA expression of genes located on amplified regions is rare [7, 10]. The aim of the present study was to investigate whether *CSE1L, DIDO1* and *RBM39* are drivers or merely passengers of 20q gain-driven colorectal adenoma-to-carcinoma progression, by investigation of mRNA and protein expression levels of these genes in colorectal adenomas and carcinomas in relation to chromosome 20q DNA copy number status.

## Materials and methods

### Correlation of mRNA expression data to DNA copy number status

Data on DNA copy number ratio of the actual gene locus and mRNA expression in colorectal adenomas and carcinomas have been previously obtained by array CGH (comparative genomic hybridization) and mRNA expression microarrays, respectively [7]. Copy number ratios of the actual gene loci were determined by taking the tumor to normal DNA copy number ratio of the BAC (bacterial artificial chromosome) clone that covers the gene locus (e.g. RP1-155G6 for *CSE1L*, RP4-563E14 for *DIDO1* and RP11-353C18 for *RBM39*). The smoothened, but not called, ratio was used as a continuous variable. Differences in DNA copy number ratio and mRNA expression levels between adenomas and carcinomas and differences between tumors with and without 20q gain were assessed by the Mann–Whitney test. Correlation of array CGH and mRNA expression data was evaluated by Pearson correlation.

### Colorectal adenoma and carcinoma tissue microarrays

Tissue microarrays had been constructed using a series of 82 colon adenomas and 82 CRCs that were collected retrospectively from 2001 to 2008 at the VU University medical center, Amsterdam, The Netherlands. All tissues were used in compliance with the institutional ethical regulations for use of patient material, in agreement with the “Code for Proper Secondary Use of Human Tissue in the Netherlands”. Tumors were characterized for MSI-status and chromosome 20q DNA copy number status as described previously [11]. Histological and molecular characteristics of the tumors are summarized in Suppl. Table 1.

### CSE1L, DIDO1 and RBM39 immunohistochemistry

Immunohistochemical stainings of CSE1L, DIDO1 and RBM39 were performed on tissue microarrays. Paraffin sections (4 μm) were deparaffinized in xylene and rehydrated through a series of graded alcohol to water. Endogenous peroxidase was blocked for 30 min with hydrogen peroxide (0.3 % H_2_O_2_ in methanol). Antigen retrieval was performed by autoclave heating in 10 mM citrate buffer (pH 6.0). Stainings were performed using CSE1L antibody (NCL-CAS, mouse monoclonal, Novocastra, Newcastle upon Tyne, UK) in a 1:30 dilution, DIDO1 antibody (sc-25264, mouse monoclonal, Santa Cruz Biotechnology, Santa Cruz, CA, USA) in a 1:100 dilution and RBM39 antibody (rabbit polyclonal, HPA001591, AtlasAntibodies in a 1:75 dilution. The HPA001591 antibody was specifically generated and used for protein profiling as part of the Human Protein Atlas project (http://www.proteinatlas.org) [12, 13]. All primary antibodies were incubated for 30 min at room temperature. Stainings were subsequently detected by HRP-coupled polymer (ThermoScientific, Warm Springs, Fremont, CA, USA) and visualized by diaminbenzidine plus (DAB Plus) (ThermoScientific). Sections were counterstained with Mayer’s haematoxylin. Incubation without primary antibody was used as negative control.

### Evaluation of protein expression and correlation to DNA copy number status

Protein expression of CSE1L, DIDO1 and RBM39 was evaluated based on staining intensity in epithelial cells and scored as weak, moderate or strong. Per tumor the maximum score of the evaluated cores was determined. For statistical analysis of protein expression, tumors were classified into two groups based on the maximum staining score; weak and moderate staining versus strong staining. The Chi-Square test was used to compare protein expression between adenomas and carcinomas and tumors with and without 20q gain. All statistical analyses were performed in SPSS (version 15.0 for Windows, SPSS, Chicago, Illinois, USA).

## Results

### Correlation of *CSE1L*, *DIDO1* and *RBM39* mRNA expression with (20q) DNA copy number and adenoma/carcinoma status

**Fig. 1:**
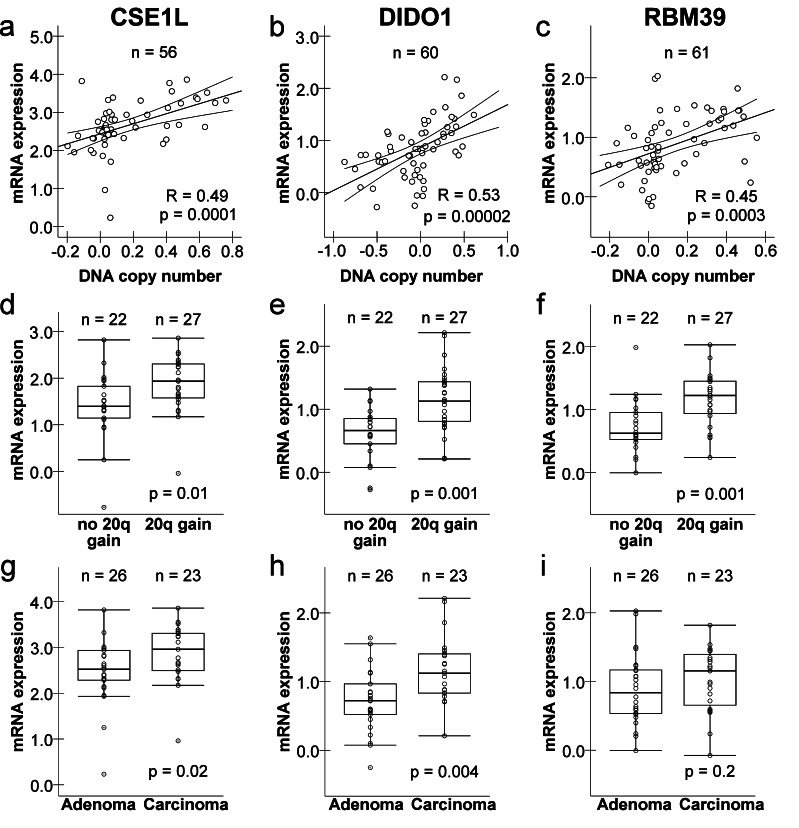
Correlation of *CSE1L*, *DIDO1* and *RBM39* mRNA expression with (20q) DNA copy number and adenoma/carcinoma status. Correlation of DNA copy number with mRNA expression for *CSE1L* **a**, *DIDO1* **b** and *RBM39* **c**. DNA copy numbers were determined by the copy number ratio of the BAC covering the gene locus. Pearson correlation coefficients and p-values are shown. Lines represent a linear regression fit with 95 % confidence intervals. *CSE1L* **d**, *DIDO1* **e** and *RBM39* **f** mRNA expression levels (arbitrary units) in tumors (*n* = 49) without and with 20q gain. P-values were determined by the Mann–Whitney test. *CSE1L* **g**, *DIDO1* **h** and *RBM39* **i** mRNA expression levels (arbitrary units) in adenomas and carcinomas for which 20q status was known (*n* = 49). P-values were determined by the Mann–Whitney test

To investigate whether *CSE1L*, *DIDO1* and *RBM39* may contribute to chromosome 20q gain-driven colorectal adenoma-to-carcinoma progression, correlation analyses were performed to examine the relationship between their DNA copy number status and mRNA expression levels. Significant correlations between DNA copy number ratios and mRNA expression levels were observed for *CSE1L* (R = 0.49 with *p* = 0.0001; Fig. 1a), *DIDO1* (R = 0.53 with *p* = 0.00002; Fig. 1b) and *RBM39* (R = 0.45 with *p* = 0.0003; Fig. 1c). Next, *CSE1L*, *DIDO1* and *RBM39* mRNA expression levels were examined in relation to chromosome 20q gain status within the group of chromosomal instable tumors. For this purpose, MSI tumors were excluded from further analysis upon which the remaining colorectal tumors (26 adenomas and 23 carcinomas) were classified into two categories based on absence or presence of 20q gain. Messenger RNA expression levels were significantly increased in tumors with 20q gain compared to tumors without 20q gain for each of these genes, i.e. *CSE1L* (*p* = 0.01; Fig. 1d), *DIDO1* (*p* = 0.001; Fig. 1e) and *RBM39* (*p* = 0.001; Fig. 1f).

Genes that are functionally relevant for tumor progression are likely to show increased mRNA expression levels in carcinomas compared to adenomas. As such, the fact that chromosome 20q gain is associated with colorectal adenoma-to-carcinoma progression prompted us to examine whether increased *CSE1L*, *DIDO1* and *RBM39* mRNA expression levels are merely associated with tumor progression or truly driven by chromosome 20q DNA copy number status. For each of these genes, the difference in median mRNA expression levels (arbitrary units) of tumors with 20q gain versus those without 20q gain (Fig. 1d-1f; *CSE1L:* 0.55, *DIDO:* 0.47, *RBM39:* 0.60) was larger than the difference in their median expression levels (arbitrary units) between those adenomas and carcinomas for which 20q status was known (*n* = 49) (Fig. 1g-i; *CSE1L:* 0.44, *DIDO:* 0.40, *RBM39:* 0.31). Results comparing adenomas to carcinomas obtained for the whole data set of 37 adenomas and 31 carcinomas for which mRNA expression data was available are shown in Suppl. Fig. 2. In fact, only the mRNA expression level of *CSE1L* (*p* = 0.02 and *DIDO1* (*p* = 0.004) remained significantly elevated in CRCs compared to adenomas. These data indicate that the mRNA expression levels of *CSE1L*, *DIDO1* and RBM39 are predominately influenced by 20q DNA copy number status rather than being generally increased due to tumor progression.

### Correlation of CSE1L, DIDO1 and RBM39 protein expression to chromosome 20q gain and adenoma/carcinoma status

**Fig. 2:**
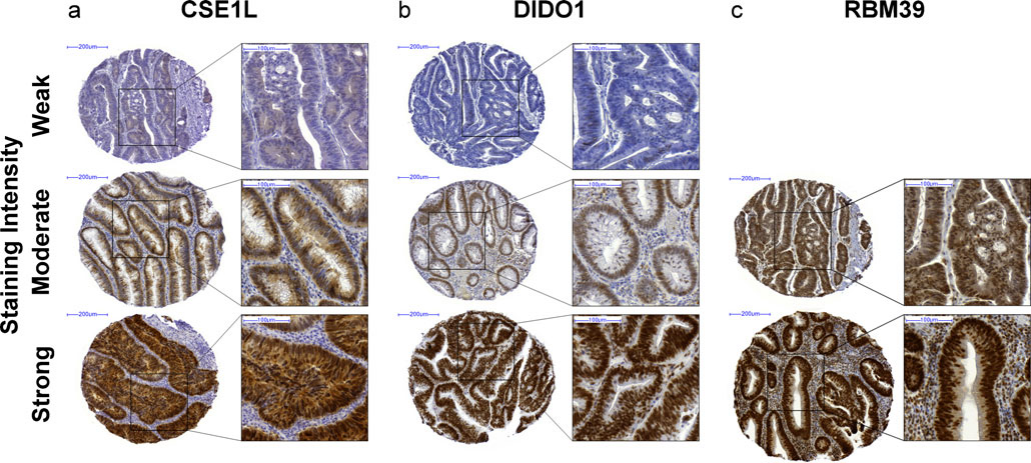
Immunohistochemical stainings of CSE1L, DIDO1 and RBM39. **a** CSE1L staining was mainly present in the nuclei of epithelial cells accompanied by a weak cytoplasmic staining. Staining intensity was analyzed based on the overall intensity in epithelial cells, and scored as weak, moderate, or strong. Representative examples of each category are shown. **b** DIDO1 staining was exclusively nuclear, and was observed in epithelial and stromal cells. Nuclear staining intensity in epithelial cells was scored as weak, moderate or strong. Representative examples of each category are shown. **c** RBM39 staining was observed in epithelial and stromal cells. Nuclear staining of RBM39 was accompanied by a faint cytoplasmic staining. Staining intensity in epithelial cells was scored as weak, moderate or strong. Due to lack of weakly stained cores, only representative examples of moderate and strong staining are shown

To investigate the correlation between chromosome 20q DNA copy number status and CSE1L, DIDO1 and RBM39 protein expression in colorectal adenoma-to-carcinoma progression, immunohistochemistry was performed on tissue microarrays containing 82 colorectal adenomas and 82 carcinomas. CSE1L protein expression was mainly present in the nuclei of epithelial cells accompanied by a weak cytoplasmic staining (Fig. 2a). DIDO1 protein expression was exclusively nuclear and observed in epithelial cells as well as stromal cells (Fig. 2b). Nuclear protein expression of RBM39 was associated with a faint cytoplasmic staining (Fig. 2c).

**Fig. 3:**
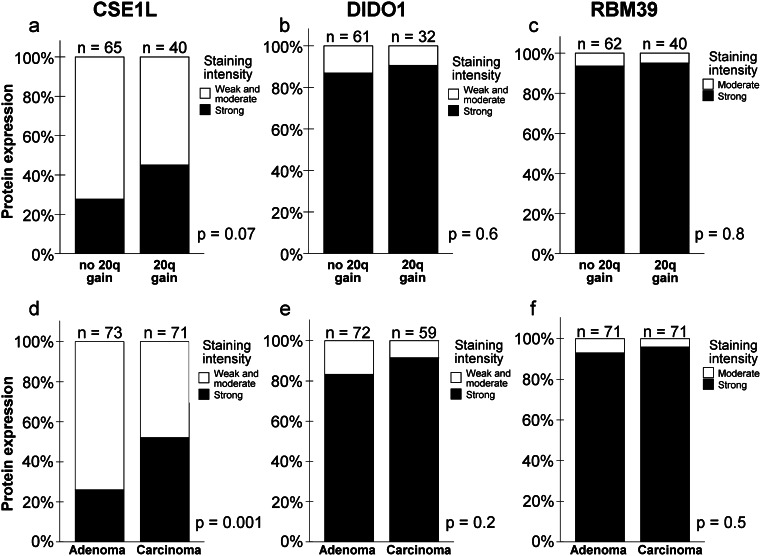
Correlation of CSE1L, DIDO1 and RBM39 protein expression with 20q DNA copy number and adenoma/carcinoma status. CSE1L **a**, DIDO1 **b** and RBM39 **c** protein expression levels in tumors without and with 20q gain. CSE1L **d**, DIDO1 **e** and RBM39 **f** protein expression levels in human colorectal adenoma and carcinoma tissues. P-values were determined using the Chi-Square test

There was no significant difference in epithelial protein expression of CSE1L, DIDO1 and RBM39 between tumors with and without chromosome 20q gain (Fig. 3a-c). However, CSE1L protein expression in epithelial cells was higher in carcinomas compared to adenomas (*p* = 0.001; Fig. 3d). Epithelial protein expression of DIDO1 and RBM39 was observed in the far majority of tumors. More than 80 % of adenomas stained strongly positive for DIDO1 and more than 90 % of adenomas for RBM39. Therefore, although the percentages of carcinomas with strong DIDO1 or RBM39 protein expression were slightly higher, significant differences could not be detected for these two proteins (Fig. 3e-f).

## Discussion

Chromosome 20q is a major factor in colorectal adenoma-to-carcinoma progression. Genes that drive 20q gain associated colorectal adenoma-to-carcinoma progression are believed to influence cancer processes due to increased DNA, mRNA and protein expression levels. The 20q amplicon harbors multiple genes that contribute to CRC carcinogenesis as indicated by the large part of the chromosome arm that is generally amplified in CRC. We recently identified *AURKA* and *TPX2* as major contributors to 20q gain associated CRC progession [9]. In the same study *CSE1L*, *DIDO1* and *RBM39* were also found to contribute to several cancer-related biological processes such as cell viability and anchorage-independent growth. To further explore the role of *CSE1L, DIDO1* and *RBM39* in colorectal adenoma-to-carcinoma progression, we here analyzed mRNA and protein levels of *CSE1L*, *DIDO1* and *RBM39* in colorectal adenomas and carcinomas in relation to 20q DNA copy number status.

### CSE1L

*CSE1L* was found to affect cell viability and anchorage-independent growth (Suppl. Fig. 1). Other studies have reported that *CSE1L* plays a role in apoptosis, chromosomal instability, migration, invasion and metastasis in different cancer types [14–20]. Increased *CSE1L* DNA copy numbers (including high level amplifications), mRNA and protein expression have been reported for CRC and other tumor types [21–26]. Overexpression of *CSE1L* has been linked to tumor progression [26–29]. In support of these published data the present study, for the first time, demonstrates increased expression of *CSE1L* mRNA and protein levels in colorectal carcinomas compared to adenomas. Based on the functional effects of *CSE1L* on cancer processes and the increased mRNA and protein levels in carcinomas compared to adenomas, *CSE1L* likely contributes to colon tumor progression. The correlation of *CSE1L* (20q) DNA copy number status with mRNA expression levels implies that transcription of *CSE1L* mRNA is influenced by chromosome 20q gain. Nevertheless, protein expression of CSE1L neither correlates with 20q DNA copy number status nor with mRNA expression levels (Suppl. Fig. 3), implying that CSE1L protein levels are subject to post-transcriptional regulation in a 20q gain-independent manner. Because CSE1L protein expression does not correlate with chromosome 20q gain status, *CSE1L* is likely a passenger rather than a driver gene of chromosome 20q gain-associated adenoma-to-carcinoma progression.

*CSE1L* (chromosome segregation 1-like, also known as Cellular Apoptosis Susceptibility—CAS – ) is a nuclear transport factor that contributes to protein transport from the cytoplasm to the nucleus and vice versa. This proces is important for normal cell function [30]. Proteins that contain a nuclear localization signal form a complex with importin-α in the cytoplasm. Subsequent binding of importin-β results in translocation of the complex to the nucleus, where the complex dissociates. Importin-β is recycled to the cytoplasm by RanGTP, whereas recycling of importin-α is mediated by CSE1L [31, 32]. The effect of CSE1L on tumor progression can be explained by its role in nuclear transport which requires nuclear localization of CSE1L [16]. The immunohistochemical stainings show this localization in colorectal tumors. Overexpression of CSE1L results in constitutive binding to importin-α, causing depletion of free importin-α which is necessary for nuclear import of proteins [30]. Aberrant expression of CSE1L may therefore cause mislocalization of cellular proteins, which can contribute to tumor development [16, 30]. Interfering with protein function by sequestering in cellular compartments has for example been described to contribute to the functional inactivation of the tumor suppressor gene *TP53* [33]. Weak CSE1L protein expression was observed in the cytoplasm of colorectal adenoma and carcinoma samples with staining levels that were directly correlated to its nuclear staining levels. Several papers emphasize (vesicular) cytoplasmic staining of CSE1L and propose a role for CSE1L in vesicle translocation and protein secretion [34–36]. Proteomics analysis of cell surface and secretomes of CRC cell lines supports the presence of CSE1L protein associated with plasma membranes and secreted microvesicles (supplementary data in de Wit et al. [11] and Fijneman et al., [37]). Cytoplasmic staining has also been described to be caused by the association of CSE1L to microtubules. Microtubule-bound CSE1L may be an inactive reservoir of CSE1L or may have a specific biological function during mitosis; e.g. in chromosome segregation, as the name indicates [38]. Chromosome segregation is a delicate process which can result in chromosomal aberrations when deregulated. A role for CSE1L in this process is in agreement with the deficiency in chromosome segregation observed in yeast cells with *CSE1L* mutations [18]. Other genes that contribute to chromosomal instability (e.g. *AURKA* and *TPX2*) have been described as playing a major role in colorectal adenoma-to-carcinoma progression [9, 39].

### DIDO1

In our previous studies, downmodulation of *DIDO1* resulted in reduced anchorage-independent growth (Suppl. Fig.1). *DIDO1* mRNA expression correlated with DNA copy number and chromosome 20q gain status (Fig. 1), which indicates that elevated *DIDO1* expression is induced by gain of the 20q amplicon. However, no differences could be detected for DIDO1 protein expression between tumors with and without chromosome 20q gain. Such lack of observed variation in protein expression may be a technical issue. Tumors without 20q gain, which have a relatively low *DIDO1* mRNA expression compared to tumors with 20q gain, already showed quite strong DIDO1 protein expression. Since immunohistochemistry is not a stoichiometric technique stainings can only be quantified within a restricted window of protein expression, upon which further increase in protein expression will not become visible as more intense staining. Therefore, the present study does not support a role for *DIDO1* as a ‘driver’ gene for adenoma-to-carcinoma progression, but rather suggests that increased *DIDO1* (mRNA) expression in 20q gain tumors results from a ‘passenger’ effect due to its localization on the 20q amplicon.

The putative functional role of DIDO1 in tumor progression is not quite clear. *DIDO1* (death inducer-obligator 1) is an early apoptosis regulator protein that has been described as a tumor suppressor gene in hematological myeloid neoplasms. Upon apoptosis induction, cytoplasmic DIDO1 translocates to the nucleus to induce caspase levels and activity [40]. Downmodulation of *DIDO1* mRNA expression, however, did not change cell viability in CRC cell lines upon 5FU-induced cytotoxicity, arguing against a role for *DIDO1* in apoptosis induction in CRC cells. Disruption of *DIDO1* has been reported to result in chromosomal instability by a defective mitotic checkpoint [41], while overexpression of *DIDO1* also induces chromosomal instability as observed by asymmetric divisions leading to the occurrence of lagging chromosomes [42]. Like *DIDO1*, inhibition of the expression of other genes with a role in chromosomal instability, such as *AURKA* and *TPX2*, also reduced anchorage-independent growth [9].

### RBM39

Downmodulation of *RBM39* expression affected cell viability in CRC cell lines (Suppl. Fig. 1). Similar to *DIDO1*, *RBM39* mRNA expression correlated with DNA copy number and chromosome 20q gain status whereas no differences could be detected in protein expression between tumors with and without chromosome 20q gain. *RBM39* mRNA and protein expression was also not different between adenomas and carcinomas. Thus, *RBM39* mRNA expression is increased due to 20q gain and could play a role in 20q gain associated tumor progression. However, the present study does not support a role for *RBM39* as a ‘driver’ gene for adenoma-to-carcinoma progression, rather suggests that increased *RBM39* (mRNA) expression in 20q gain tumors results from a ‘passenger’ effect due to its localization on the 20q amplicon.

*RBM39* (RNA binding motif protein 39, also known as CAPER alpha) is a transcriptional coactivator that stimulates transcription mediated by the progesteron and estrogen steroid hormone receptors. In addition, RBM39 contributes to alternative splicing, which is an important mechanism to create mRNA, protein and functional diversity by modulation of processing of pre-mRNAs [43, 44]. Alterations in the activity of proteins involved in alternative splicing have been described to contribute to tumor development and progression, e.g. for AURKA and TPX2 [45–47]. *RBM39* has been described to be overexpressed in small-cell lung cancer and breast cancer [48, 49]. In breast cancer a shift in RBM39 expression was observed from the cytoplasm to the nucleus during transition from pre-malignancy to carcinoma in situ [49]. Such a shift in subcellular localization of RBM39 protein was not observed for the transition of colorectal adenomas into carcinomas in our experiments, since pre-malignant adenomas already showed strong nuclear staining.

### Summary

In conclusion, *CSE1L*, *DIDO1* and *RBM39* can influence tumor progression by their functional role in various cancer processes. However, despite their significant correlation between DNA copy number status and mRNA expression levels, lack of correlation to protein expression levels does not support these genes as ‘drivers’ for 20q gain associated adenoma-to-carcinoma progression. Instead, their increased mRNA expression appears to be a ‘passenger’ effect due to their localization on the 20q gain amplicon.

## Declarations

Collection, storage, and use of tissue and patient data were performed in agreement with the ‘Code for Proper Secondary Use of Human Tissue in the Netherlands’, in compliance with national and institutional ethical regulations. The authors declare that they have no conflict of interest.

## Electronic supplementary material

Below is the link to the electronic supplementary material.

Suppl. Table 1 Histological and molecular characteristics of 164 colorectal tumors on the tissue microarray (DOC 50 kb)

ESM 1 (DOC 839 kb)

## References

[1] A. Jemal, R. Siegel, E. Ward, Y. Hao, J. Xu, M.J. Thun, Cancer statistics, 2009. CA Cancer J. Clin. **59**, 225 (2009) 19474385 10.3322/caac.20006

[2] H. Shinya, W.I. Wolff, Morphology, anatomic distribution and cancer potential of colonic polyps. Ann. Surg. **190**, 679 (1979) 518167 10.1097/00000658-197912000-00001 PMC1345622

[3] H. Rajagopalan, C. Lengauer, Aneuploidy and cancer. Nature **432**, 338 (2004) 15549096 10.1038/nature03099

[4] A. Esquela-Kerscher, F.J. Slack, Oncomirs—microRNAs with a role in cancer. Nat. Rev. Cancer **6**, 259 (2006) 16557279 10.1038/nrc1840

[5] A.H. Sillars-Hardebol, B. Carvalho, M. van Engeland, R.J. Fijneman, G.A. Meijer, The adenoma hunt in colorectal cancer screening: defining the target. J. Pathol. **226**, 1 (2012) 21984228 10.1002/path.3012

[6] P.M. De Angelis, O.P. Clausen, A. Schjolberg, T. Stokke, Chromosomal gains and losses in primary colorectal carcinomas detected by CGH and their associations with tumour DNA ploidy, genotypes and phenotypes. Br. J. Cancer **80**, 526 (1999) 10408863 10.1038/sj.bjc.6690388 PMC2362312

[7] B. Carvalho, C. Postma, S. Mongera, E. Hopmans, S. Diskin, M.A. van de Wiel, W. van Criekinge, O. Thas, A. Matthai, M.A. Cuesta, J.S. Terhaar Sive Droste, M. Craanen, E. Schrock, B. Ylstra, G.A. Meijer, Multiple putative oncogenes at the chromosome 20q amplicon contribute to colorectal adenoma to carcinoma progression. Gut **58**, 79 (2009) 18829976 10.1136/gut.2007.143065

[8] C. Postma, S. Terwischa, M.A. Hermsen, J.R. van der Sijp, G.A. Meijer, Gain of chromosome 20q is an indicator of poor prognosis in colorectal cancer. Cell. Oncol. **29**, 73 (2007) 17429145 10.1155/2007/137592 PMC4617993

[9] A.H. Sillars-Hardebol, B. Carvalho, M. Tijssen, J.A. Belien, M. de Wit, P.M. Delis-van Diemen, F. Ponten, M.A. van de Wiel, R.J. Fijneman, G.A. Meijer, TPX2 and AURKA promote 20q amplicon-driven colorectal adenoma to carcinoma progression. Gut doi:10.1136/gutjnl-2011-301153 (Epub ahead of print) 10.1136/gutjnl-2011-301153 22207630

[10] P. Platzer, M.B. Upender, K. Wilson, J. Willis, J. Lutterbaugh, A. Nosrati, J.K. Willson, D. Mack, T. Ried, S. Markowitz, Silence of chromosomal amplifications in colon cancer. Cancer Res. **62**, 1134 (2002) 11861394

[11] M. de Wit, C.R. Jimenez, B. Carvalho, J.A. Belien, P.M. Delis-van Diemen, S. Mongera, S.R. Piersma, M. Vikas, S. Navani, F. Ponten, G.A. Meijer, R.J. Fijneman, Cell surface proteomics identifies glucose transporter type 1 and prion protein as candidate biomarkers for colorectal adenoma-to-carcinoma progression. Gut **61**, 855 (2012) 21890811 10.1136/gutjnl-2011-300511

[12] M. Uhlen, E. Bjorling, C. Agaton, C.A. Szigyarto, B. Amini, E. Andersen, A.C. Andersson, P. Angelidou, A. Asplund, C. Asplund, L. Berglund, K. Bergstrom, H. Brumer, D. Cerjan, M. Ekstrom, A. Elobeid, C. Eriksson, L. Fagerberg, R. Falk, J. Fall, M. Forsberg, M.G. Bjorklund, K. Gumbel, A. Halimi, I. Hallin, C. Hamsten, M. Hansson, M. Hedhammar, G. Hercules, C. Kampf, K. Larsson, M. Lindskog, W. Lodewyckx, J. Lund, J. Lundeberg, K. Magnusson, E. Malm, P. Nilsson, J. Odling, P. Oksvold, I. Olsson, E. Oster, J. Ottosson, L. Paavilainen, A. Persson, R. Rimini, J. Rockberg, M. Runeson, A. Sivertsson, A. Skollermo, J. Steen, M. Stenvall, F. Sterky, S. Stromberg, M. Sundberg, H. Tegel, S. Tourle, E. Wahlund, A. Walden, J. Wan, H. Wernerus, J. Westberg, K. Wester, U. Wrethagen, L.L. Xu, S. Hober, F. Ponten, A human protein atlas for normal and cancer tissues based on antibody proteomics. Mol. Cell Proteomics. **4**, 1920 (2005) 16127175 10.1074/mcp.M500279-MCP200

[13] M. Uhlen, P. Oksvold, L. Fagerberg, E. Lundberg, K. Jonasson, M. Forsberg, M. Zwahlen, C. Kampf, K. Wester, S. Hober, H. Wernerus, L. Bjorling, F. Ponten, Towards a knowledge-based Human Protein Atlas. Nat. Biotechnol. **28**, 1248 (2010) 21139605 10.1038/nbt1210-1248

[14] U. Brinkmann, E. Brinkmann, M. Gallo, I. Pastan, Cloning and characterization of a cellular apoptosis susceptibility gene, the human homologue to the yeast chromosome segregation gene CSE1. Proc. Natl. Acad. Sci. U. S. A. **92**, 10427 (1995) 7479798 10.1073/pnas.92.22.10427 PMC40810

[15] U. Brinkmann, E. Brinkmann, M. Gallo, U. Scherf, I. Pastan, Role of CAS, a human homologue to the yeast chromosome segregation gene CSE1, in toxin and tumor necrosis factor mediated apoptosis. Biochemistry **35**, 6891 (1996) 8639641 10.1021/bi952829+

[16] U. Brinkmann, CAS, the human homologue of the yeast chromosome-segregation gene CSE1, in proliferation, apoptosis, and cancer. Am. J. Hum. Genet. **62**, 509 (1998) 9497270 10.1086/301773 PMC1376967

[17] A. Wellmann, L. Krenacs, T. Fest, U. Scherf, I. Pastan, M. Raffeld, U. Brinkmann, Localization of the cell proliferation and apoptosis-associated CAS protein in lymphoid neoplasms. Am. J. Pathol. **150**, 25 (1997) 9006318 PMC1858504

[18] Z. Xiao, J.T. McGrew, A.J. Schroeder, M. Fitzgerald-Hayes, CSE1 and CSE2, two new genes required for accurate mitotic chromosome segregation in Saccharomyces cerevisiae. Mol. Cell. Biol. **13**, 4691 (1993) 8336709 10.1128/mcb.13.8.4691-4702.1993 PMC360095

[19] C.F. Liao, S.F. Luo, L.T. Li, C.Y. Lin, Y.C. Chen, M.C. Jiang, CSE1L/CAS, the cellular apoptosis susceptibility protein, enhances invasion and metastasis but not proliferation of cancer cells. J. Exp. Clin. Cancer Res. **27**, 15 (2008) 18597698 10.1186/1756-9966-27-15 PMC2474842

[20] C.J. Tai, S.C. Shen, W.R. Lee, C.F. Liao, W.P. Deng, H.Y. Chiou, C.I. Hsieh, J.N. Tung, C.S. Chen, J.F. Chiou, L.T. Li, C.Y. Lin, C.H. Hsu, M.C. Jiang, Increased cellular apoptosis susceptibility (CSE1L/CAS) protein expression promotes protrusion extension and enhances migration of MCF-7 breast cancer cells. Exp. Cell Res. **316**, 2969 (2010) 20688056 10.1016/j.yexcr.2010.07.019

[21] U. Brinkmann, M. Gallo, M.H. Polymeropoulos, I. Pastan, The human CAS (cellular apoptosis susceptibility) gene mapping on chromosome 20q13 is amplified in BT474 breast cancer cells and part of aberrant chromosomes in breast and colon cancer cell lines. Genome Res. **6**, 187 (1996) 8963895 10.1101/gr.6.3.187

[22] A.B. Hui, K.W. Lo, P.M. Teo, K.F. To, D.P. Huang, Genome wide detection of oncogene amplifications in nasopharyngeal carcinoma by array based comparative genomic hybridization. Int. J. Oncol. **20**, 467 (2002) 11836556

[23] C.Y. Tong, A.B. Hui, X.L. Yin, J.C. Pang, X.L. Zhu, W.S. Poon, H.K. Ng, Detection of oncogene amplifications in medulloblastomas by comparative genomic hybridization and array-based comparative genomic hybridization. J. Neurosurg. **100**, 187 (2004) 14758948 10.3171/ped.2004.100.2.0187

[24] H. Brustmann, Expression of cellular apoptosis susceptibility protein in serous ovarian carcinoma: a clinicopathologic and immunohistochemical study. Gynecol. Oncol. **92**, 268 (2004) 14751170 10.1016/j.ygyno.2003.10.029

[25] K. Shiraki, K. Fujikawa, K. Sugimoto, T. Ito, T. Yamanaka, M. Suzuki, K. Yoneda, K. Sugimoto, K. Takase, T. Nakano, Cellular apoptosis susceptibility protein and proliferation in human hepatocellular carcinoma. Int. J. Mol. Med. **18**, 77 (2006) 16786158

[26] I.M. Seiden-Long, K.R. Brown, W. Shih, D.A. Wigle, N. Radulovich, I. Jurisica, M.S. Tsao, Transcriptional targets of hepatocyte growth factor signaling and Ki-ras oncogene activation in colorectal cancer. Oncogene **25**, 91 (2006) 16158056 10.1038/sj.onc.1209005

[27] A. Wellmann, P. Flemming, P. Behrens, K. Wuppermann, H. Lang, K. Oldhafer, I. Pastan, U. Brinkmann, High expression of the proliferation and apoptosis associated CSE1L/CAS gene in hepatitis and liver neoplasms: Correlation with tumor progression. Int. J. Mol. Med. **7**, 489 (2001) 11295109 10.3892/ijmm.7.5.489

[28] P. Behrens, U. Brinkmann, F. Fogt, N. Wernert, A. Wellmann, Implication of the proliferation and apoptosis associated CSE1L/CAS gene for breast cancer development. Anticancer. Res. **21**, 2413 (2001) 11724300

[29] G. Peiro, J. Diebold, G.B. Baretton, R. Kimmig, U. Lohrs, Cellular apoptosis susceptibility gene expression in endometrial carcinoma: Correlation with Bcl-2, Bax, and caspase-3 expression and outcome. Int. J. Gynecol. Pathol. **20**, 359 (2001) 11603220 10.1097/00004347-200110000-00008

[30] T.R. Kau, J.C. Way, P.A. Silver, Nuclear transport and cancer: From mechanism to intervention. Nat. Rev. Cancer **4**, 106 (2004) 14732865 10.1038/nrc1274

[31] U. Kutay, F.R. Bischoff, S. Kostka, R. Kraft, D. Gorlich, Export of importin alpha from the nucleus is mediated by a specific nuclear transport factor. Cell **90**, 1061 (1997) 9323134 10.1016/s0092-8674(00)80372-4

[32] K.S. Ullman, M.A. Powers, D.J. Forbes, Nuclear export receptors: From importin to exportin. Cell **90**, 967 (1997) 9323123 10.1016/s0092-8674(00)80361-x

[33] K.M. Ryan, A.C. Phillips, K.H. Vousden, Regulation and function of the p53 tumor suppressor protein. Curr. Opin. Cell Biol. **13**, 332 (2001) 11343904 10.1016/s0955-0674(00)00216-7

[34] W.C. Uen, C.J. Tai, S.C. Shen, W.R. Lee, T.Y. Tsao, W.P. Deng, H.Y. Chiou, C.H. Hsu, C.I. Hsieh, C.F. Liao, M.C. Jiang, Differential distributions of CSE1L/CAS and E-cadherin in the polarized and non-polarized epithelial glands of neoplastic colorectal epithelium. J. Mol. Histol. **41**, 259 (2010) 20734115 10.1007/s10735-010-9286-2

[35] C.S. Stella Tsai, H.C. Chen, J.N. Tung, S.S. Tsou, T.Y. Tsao, C.F. Liao, Y.C. Chen, C.Y. Yeh, K.T. Yeh, M.C. Jiang, Serum cellular apoptosis susceptibility protein is a potential prognostic marker for metastatic colorectal cancer. Am. J. Pathol. **176**, 1619 (2010) 20150437 10.2353/ajpath.2010.090467 PMC2843454

[36] T.Y. Tsao, C.S. Tsai, J.N. Tung, S.L. Chen, C.H. Yue, C.F. Liao, C.C. Wang, M.C. Jiang, Function of CSE1L/CAS in the secretion of HT-29 human colorectal cells and its expression in human colon. Mol. Cell. Biochem. **327**, 163 (2009) 19224336 10.1007/s11010-009-0054-0

[37] R.J. Fijneman, M. de Wit, M. Pourghiasian, S.R. Piersma, T.V. Pham, M.O. Warmoes, M. Lavaei, C. Piso, F. Smit, P.M. Delis-van Diemen, S.T. van Turenhout, J.S.Terhaar sive Droste, C.J. Mulder, M.A. Blankenstein, E.C. Robanus-Maandag, R. Smits, R. Fodde, V.W. van Hinsbergh, G.A. Meijer, C.R. Jimenez, Proximal fluid proteome profiling of mouse colon tumors reveals biomarkers for early diagnosis of human colorectal cancer. Clin. Cancer Res. **18**, 2613 (2012) 22351690 10.1158/1078-0432.CCR-11-1937

[38] U. Scherf, I. Pastan, M.C. Willingham, U. Brinkmann, The human CAS protein which is homologous to the CSE1 yeast chromosome segregation gene product is associated with microtubules and mitotic spindle. Proc. Natl. Acad. Sci. U. S. A. **93**, 2670 (1996) 8610099 10.1073/pnas.93.7.2670 PMC39688

[39] A.H. Sillars-Hardebol, B. Carvalho, M. de Wit, C. Postma, P.M. Delis-van Diemen, S. Mongera, B. Ylstra, M.A. van de Wiel, G.A. Meijer, R.J. Fijneman, Identification of key genes for carcinogenic pathways associated with colorectal adenoma-to-carcinoma progression. Tumour. Biol. **31**, 89 (2010) 20358421 10.1007/s13277-009-0012-1 PMC2848338

[40] D. Garcia-Domingo, D. Ramirez, G. Gonzalez de Buitrago, A. Martinez, Death inducer-obliterator 1 triggers apoptosis after nuclear translocation and caspase upregulation. Mol. Cell. Biol. **23**, 3216 (2003) 12697821 10.1128/MCB.23.9.3216-3225.2003 PMC153187

[41] V. Trachana, K.H. van Wely, A.A. Guerrero, A. Futterer, A. Martinez, Dido disruption leads to centrosome amplification and mitotic checkpoint defects compromising chromosome stability. Proc. Natl. Acad. Sci. U. S. A. **104**, 2691 (2007) 17299043 10.1073/pnas.0611132104 PMC1815243

[42] A.M. Rojas, L. Sanchez-Pulido, A. Futterer, K.H. van Wely, A. Martinez, A. Valencia, Death inducer obliterator protein 1 in the context of DNA regulation. Sequence analyses of distant homologues point to a novel functional role. FEBS J. **272**, 3505 (2005) 16008551 10.1111/j.1742-4658.2005.04759.x

[43] D.J. Jung, S.Y. Na, D.S. Na, J.W. Lee, Molecular cloning and characterization of CAPER, a novel coactivator of activating protein-1 and estrogen receptors. J. Biol. Chem. **277**, 1229 (2002) 11704680 10.1074/jbc.M110417200

[44] D.H. Dowhan, E.P. Hong, D. Auboeuf, A.P. Dennis, M.M. Wilson, S.M. Berget, B.W. O’Malley, Steroid hormone receptor coactivation and alternative RNA splicing by U2AF65-related proteins CAPERalpha and CAPERbeta. Mol. Cell **17**, 429 (2005) 15694343 10.1016/j.molcel.2004.12.025

[45] A. Srebrow, A.R. Kornblihtt, The connection between splicing and cancer. J. Cell Sci. **119**, 2635 (2006) 16787944 10.1242/jcs.03053

[46] J.P. Venables, Unbalanced alternative splicing and its significance in cancer. Bioessays **28**, 378 (2006) 16547952 10.1002/bies.20390

[47] M.J. Moore, Q. Wang, C.J. Kennedy, P.A. Silver, An alternative splicing network links cell-cycle control to apoptosis. Cell **142**, 625 (2010) 20705336 10.1016/j.cell.2010.07.019 PMC2924962

[48] C.S. Bangur, A. Switzer, L. Fan, M.J. Marton, M.R. Meyer, T. Wang, Identification of genes over-expressed in small cell lung carcinoma using suppression subtractive hybridization and cDNA microarray expression analysis. Oncogene **21**, 3814 (2002) 12032850 10.1038/sj.onc.1205480

[49] I. Mercier, M.C. Casimiro, J. Zhou, C. Wang, C. Plymire, K.G. Bryant, K.M. Daumer, F. Sotgia, G. Bonuccelli, A.K. Witkiewicz, J. Lin, T.H. Tran, J. Milliman, P.G. Frank, J.F. Jasmin, H. Rui, R.G. Pestell, M.P. Lisanti, Genetic ablation of caveolin-1 drives estrogen-hypersensitivity and the development of DCIS-like mammary lesions. Am. J. Pathol. **174**, 1172 (2009) 19342371 10.2353/ajpath.2009.080882 PMC2671351

