# Supplementary material for: CSE1L, DIDO1 and RBM39 in colorectal adenoma to carcinoma progression
Source: Cell Oncol (Dordr). 2012 Jun 19;35(4):293–300. doi: 10.1007/s13402-012-0088-2 (PMC12994943; doi:10.1007/s13402-012-0088-2)
Supplement: Supplementary file 1 — Histological and molecular characteristics of 164 colorectal tumors on the tissue microarray (DOC 50 kb) [file 13402_2012_88_MOESM1_ESM.doc]

**Suppl. Table 1** Histological and molecular characteristics of 164 colorectal tumors on the tissue microarray

|  |  |  |  |  |  |
| --- | --- | --- | --- | --- | --- |
|  |  | Number |  |  | Number |
|  |  |  |  |  |  |
| **Lesion** | **Adenoma** | 82 |  | **Carcinoma** | 82 |
|  |  |  |  |  |  |
|  |  |  |  |  |  |
| **Histological type** | Tubular | 46 | **Differentiation grade** | Poor | 10 |
|  | Tubulovillous | 33 |  | Moderate | 70 |
|  | Villous | 3 |  | Well | 2 |
|  |  |  |  |  |  |
|  |  |  |  |  |  |
| **Dysplasia** | Mild | 10 | **Dukes stage** | A | 30 |
|  | Moderate | 60 |  | B | 32 |
|  | Severe | 12 |  | C | 18 |
|  |  |  |  | D | 2 |
|  |  |  |  |  |  |
|  |  |  |  |  |  |
| **Microsatellite instable** | MSS | 73 | **Microsatellite instable** | MSS | 65 |
| n = 75^a^ | MSI | 2 | n = 76^a^ | MSI | 11 |
|  |  |  |  |  |  |
|  |  |  |  |  |  |
| **20q status of MSS tumors** n = 65^a^ | 20q gain | 9 | **20q status of MSS tumors** n = 55^a^ | 20q gain | 37 |
|  | no 20q gain | 56 |  | no 20q gain | 18 |
|  |  |  |  |  |  |

^a^ Data available for limited number of samples due to technical difficulties

MSS = microsatellite stable, MSI = microsatellite instable
