# Supplementary material for: CSE1L, DIDO1 and RBM39 in colorectal adenoma to carcinoma progression
Source: Cell Oncol (Dordr). 2012 Jun 19;35(4):293–300. doi: 10.1007/s13402-012-0088-2 (PMC12994943; doi:10.1007/s13402-012-0088-2)
Supplement: Supplementary file 2 — (DOC 839 kb) [file 13402_2012_88_MOESM2_ESM.doc]

**Suppl. Fig. 1**

**
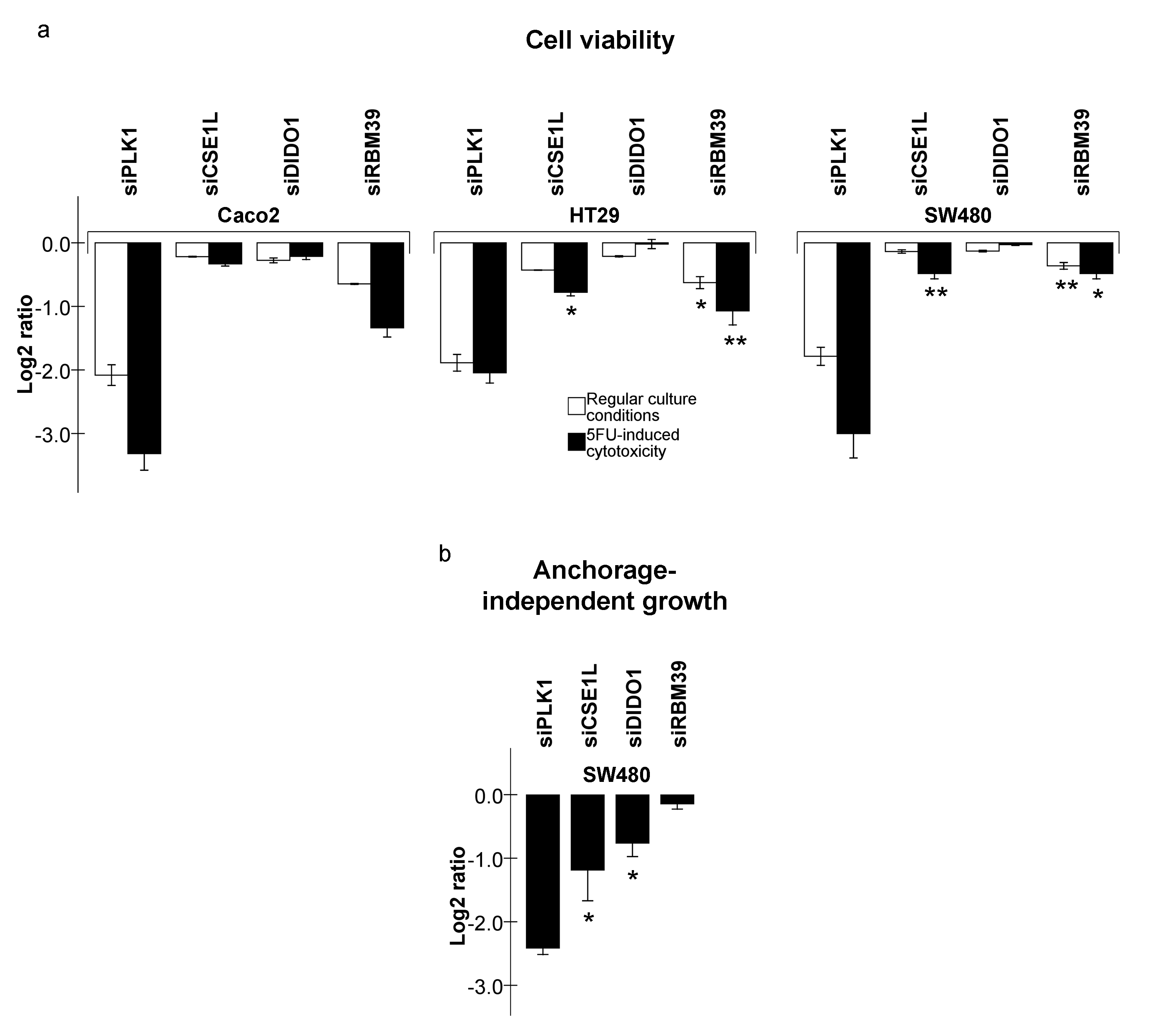
**

**Suppl. Fig. 1** Effects of downmodulation of *CSE1L*, *DIDO1* and *RBM39* on cancer-related processes. **a** Effect of siCSE1L, siDIDO1 and siRBM39 on cell viability in Caco2, HT29 and SW480 CRC cell lines under regular culture conditions and with 5FU-induced cytotoxicity as measured by the MTT cell viability assay. Experiments were performed in triplicate. siPLK1 was used as a positive control. PLK1 is a cell cycle regulator which, when downregulated, has dramatic effects on cell viability and is therefore used as positive control in genome-wide siRNA screens [1, 2]. **b** Effect of siCSE1L, siDIDO1 and siRBM39 on anchorage-independent growth as measured by the number of colonies formed in soft agarose. Three independent experiments were performed. siPLK1 was used as a positive control. Bars represent the normalized log2 ratio of cells transfected with siRNAs directed against *CSE1L*, *DIDO1*, *RBM39* and positive control genes relative to siNON-TARGETING cells (set to 0). Error bars correspond to standard errors. *P* values were determined using a permutation-rank test for negative outliers: * *P* < 0.05 and ** *P* < 0.01

1. X. Liu, R.L. Erikson, Polo-like kinase (Plk)1 depletion induces apoptosis in cancer cells. Proc. Natl. Acad. Sci. U. S. A **100**, 5789 (2003)

2. M.R. Schlabach, J. Luo, N.L. Solimini, G. Hu, Q. Xu, M.Z. Li, Z. Zhao, A. Smogorzewska, M.E. Sowa, X.L. Ang, T.F. Westbrook, A.C. Liang, K. Chang, J.A. Hackett, J.W. Harper, G.J. Hannon, S.J. Elledge, Cancer proliferation gene discovery through functional genomics. Science **319**, 620 (2008)

**Suppl. Fig. 2**

**
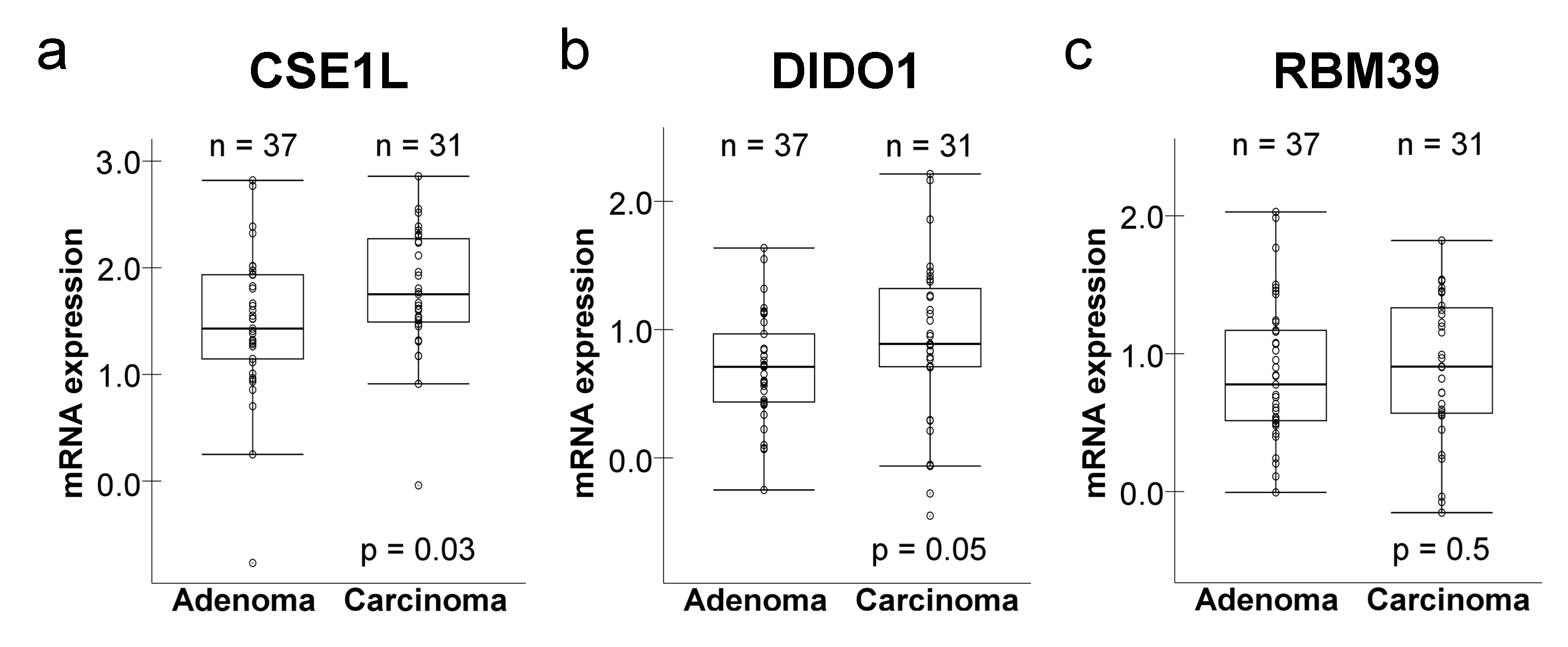
**

**Suppl. Fig. 2** Correlation of *CSE1L*, *DIDO1* and *RBM39* mRNA expression with adenoma/carcinoma status. *CSE1L* (**a**), *DIDO1* (**b**) and *RBM39* (**c**) mRNA expression levels (arbitrary units) in the complete set of adenomas and carcinomas for which mRNA expression data was available. P-values were determined by the Mann-Whitney test

**Suppl. Fig. 3**

**
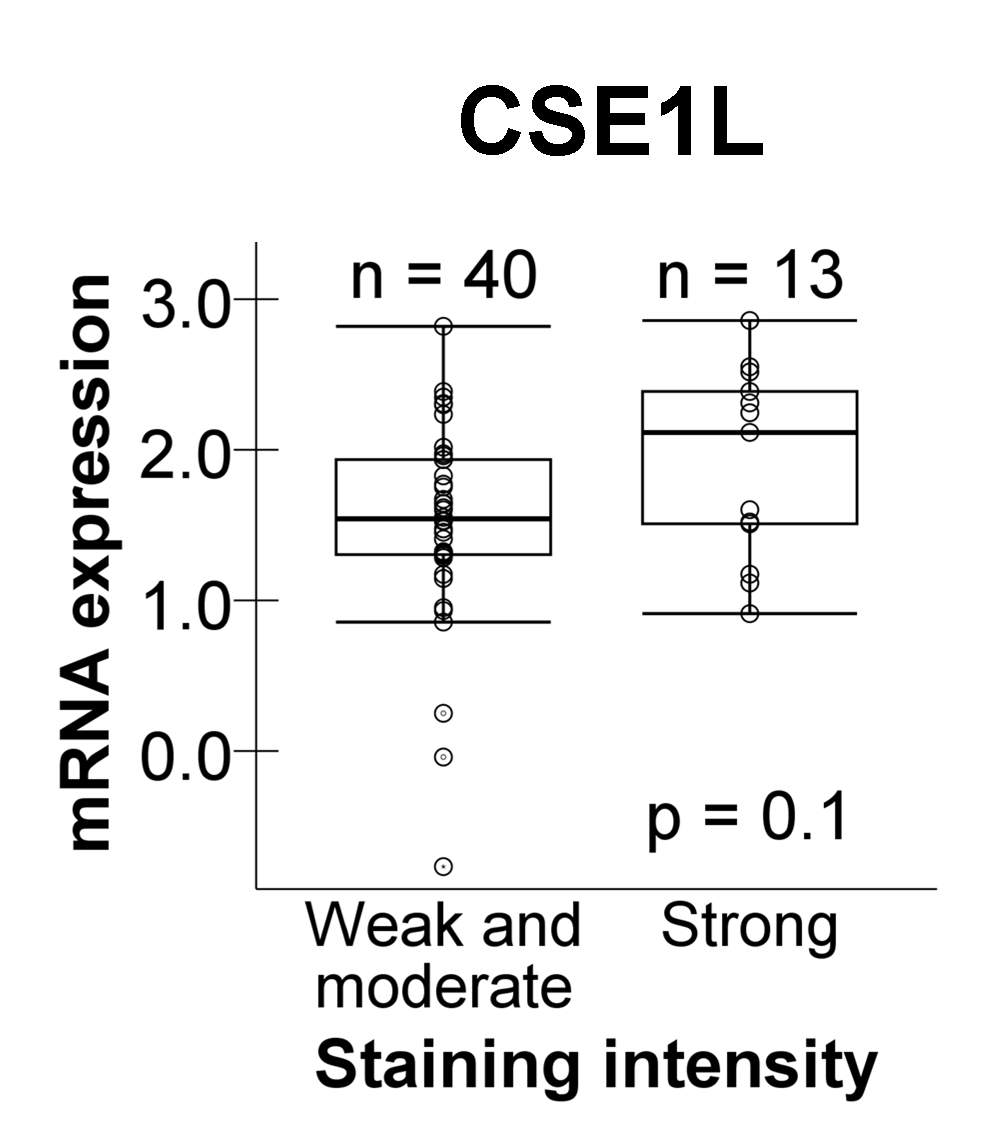
**

**Suppl. Fig. 3** Correlation of *CSE1L* mRNA expression with protein expression. P-values were determined by the Mann-Whitney test
